# Mapping Trends Regarding the Cardiopulmonary Resuscitation: A Bibliometric Analysis of the Top 100 Cited Articles

**DOI:** 10.1155/emmi/9975595

**Published:** 2025-01-15

**Authors:** Ömer Faruk Turan, Ali Sami Yardımcı, Merve Yazla, Nurullah İshak Işık, Abdullah Osman Koçak, Burak Katipoğlu

**Affiliations:** ^1^Department of Emergency Medicine, Etlik City Hospital, Ankara, Turkey; ^2^Department of Emergency Medicine, Balikesir Ataturk City Hospital, Balikesir, Turkey

**Keywords:** bibliometrics, cardiac arrest, cardiopulmonary resuscitation, Scopus

## Abstract

**Introduction:** Despite significant medical and technological advancements, cardiac arrest remains a critical public health concern due to its persistently high mortality and morbidity rates. Consequently, research on cardiopulmonary resuscitation (CPR) is of significant importance.

**Materials and Methods:** This study presents a bibliometric analysis of the 100 most-cited articles in the field of CPR identified using the Scopus database without time restrictions. Analyses were conducted using VOSviewer and Bibliometrix software. Original research articles published in English were included.

**Results:** The study revealed contributions from 798 distinct authors across 18 journals, with citation counts ranging from 218 least-cited articles to 1194 most-cited articles. The most recent article was published in 2020, and the most frequently used keywords were “CPR” and “heart arrest.” In addition, 20% of the analyzed articles were funded by military organizations.

**Conclusion:** This analysis highlights the significant contributions of emergency medicine researchers and the notable development of CPR literature, particularly between the late 1990s and the early 2000s. While the most-cited studies originated from journals with high H-indices, the citation frequency of these articles showed a consistent decline over time. Furthermore, impactful articles in this field were predominantly published in general medical journals rather than in specialized emergency medicine journals. This study provides a foundational resource for researchers, especially early career academicians, seeking to engage in CPR-related research.

## 1. Introduction

Cardiac arrest represents a significant public health challenge because of its high rates of mortality and morbidity, which are often linked to various causes, particularly coronary heart disease [1]. Cardiopulmonary resuscitation (CPR) is a critical intervention aimed at restoring circulatory and respiratory functions in patients who lack spontaneous respiration and circulation. CPR encompasses multiple components including chest compressions, oxygen delivery, intravenous access, and medication administration. Despite substantial medical and technological advancements in the 21st century, survival rates after cardiac arrest remain below 10% in several regions worldwide [1, 2]. This persistently low survival rate underscores the ongoing need for research and updates on CPR practices. Regardless of specialty, all physicians must be proficient in cardiac arrest management and remain informed about the latest advancements in upholding their right to life.

The number of citations an article receives is often considered an indicator of its acceptance and value in the scientific community. Scientific tradition requires authors to reference prior studies relevant to their work to ensure proper documentation and acknowledgment of previous contributions [3, 4]. Bibliometrics is a widely used method that objectively and quantitatively evaluates scientific output using criteria, such as citation count, h-index, and journal impact factors. This approach seeks to answer questions such as, “What is the impact of an article post-publication?” “How can an article's influence be measured?” [3]. Advanced software facilitates the power analysis of articles, offers insights into under-researched areas, guides new research strategies, identifies trends and impacts within existing studies, and assists funding institutions in allocating resources. Bibliometric analysis also allows for the comparison of research standards among authors and contributes to the advancement of science through original contributions [4, 5]. The increasing importance of these analyses reflects their utility for assessing the reliability, quality, and influence of published works [6, 7]. By focusing on specialized fields, bibliometric analyses provide a snapshot of existing knowledge, highlighting areas that require further investigation and innovation.

In this study, we conducted a bibliometric analysis of the original articles on CPR using the Scopus database. We examined the most cited studies in cardiac arrest management, current research trends, and the most prolific authors and journals, and analyzed their interrelationships. Through this analysis, we identified historical trends, regional patterns, and key topics by focusing on the 100 most cited publications. Our aim is to provide a comprehensive guide for researchers seeking to conduct impactful studies and achieve high citation rates.

## 2. Materials and Methods

A literature review was conducted by two independent emergency medicine physicians, with a third consulting physician to resolve any disagreements. Throughout this study, the Scopus database (https://www.scopus.com/) was used as the primary data source. Numerous studies have compared databases such as Scopus, PubMed (https://pubmed.ncbi.nlm.nih.gov), Web of Science (WOS) (https://www.webofscience.com/), and Google Scholar (https://scholar.google.com/) for analysis purposes [8, 9]. Scopus offers a broader search network than WOS and is more reliable than Google Scholar [10]. PubMed, in contrast, does not provide citation-based ranking or analysis capabilities. Considering these factors, including an extensive publication pool, advanced analytical tools, and high reliability supported by previous bibliometric studies, Scopus was chosen as the database for our analysis [11–13]. Article retrieval for this study was completed on March 6, 2024.

A search was conducted using commonly used keywords across all fields: “Emergency Medicine” AND “CPR” OR “Cardiopulmonary resuscitation” OR “Chest compressions” OR “Cardiac massage” OR “Heart massage” OR “Basic Cardiac Life Support” OR “Code Blue” OR “Mouth-to-mouth resuscitation” OR “Life support” OR “Heart arrest.” These keywords were identified using the Medical Subject Headings (MeSH) database (https://www.nlm.nih.gov/mesh/meshhome.html). As emergency physicians are the primary practitioners of CPR, this study focused on articles related to emergency medicine.

The first author's country and institution were recorded as articles with multiple authors from different countries and institutions. The impact factors of the journals were obtained from the Web of Science 2022 Journal Citation Reports (https://jcr.clarivate.com), ensuring a reliance on highly reputable sources. Bibliometric analyses and visualizations were performed using VOSviewer 1.6.19 (Leiden University, Netherlands), the online Bibliometrix platform (https://bibliometrix.com), and the WOS online analysis tool, with a focus on cocitation data.

Inclusion criteria are as follows:

1. Articles published in journals indexed in the Web of Science Thomson Reuters database: Science Citation Index Expanded (SCIE) and Social Sciences Citation Index (SSCI).
2. Original research articles (open access or subscription based).
3. Articles retrieved through the specified keyword search.
4. Articles written in English.

Exclusion criteria are as follows:

1. Article types other than original research articles (such as reviews, editorials, and case reports).
2. Articles without full-text availability.
3. Articles related to CPR for patients aged < 18 years.

**Figure 1:**
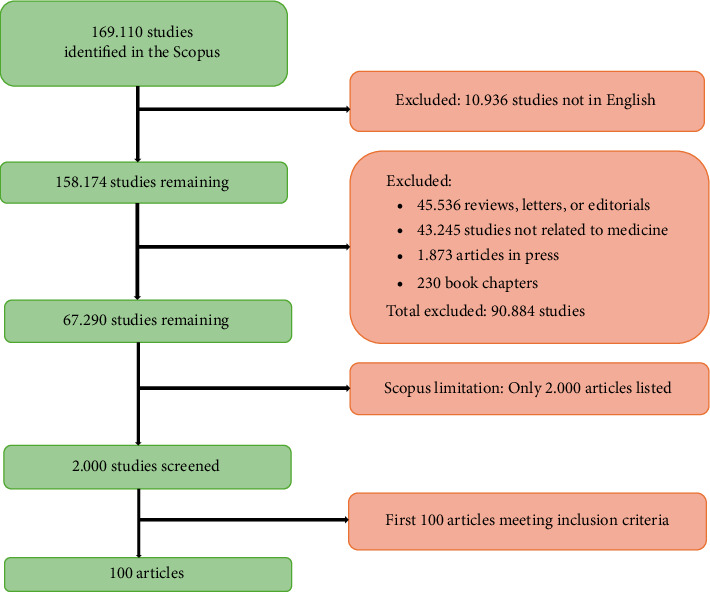
Flow diagram of the inclusion process.

Irrelevant articles were manually filtered, and the specific processes for enrollment and selection are illustrated in Figure 1.

Although we used the Scopus database as the primary source, the same search was conducted using the WOS database. No additions or exclusions were identified in the top 100 list, with only variations in citation counts and rankings. This consistency indicates that the articles we identified were highly ranked across different indices, reflecting the rigorous and meticulous nature of our analytical approach. Although non-English articles were excluded from our analysis, repeating the same search without language restrictions did not yield any changes to our list. Remarkably, all the top 100 articles were written in English, even in the absence of language limitations.

## 3. Results

A total of 798 authors from 18 different journals contributed to the creation of the 100 most cited articles. Each article had an average of 10 co-authors, with 32% of the articles involving international collaborations. The average age of the articles was 19.6 years, with the most recent article published in 2020. The average number of citations per article is 418, with citation counts ranging from 218 for the least cited articles to 1194 for the most cited articles.

**Figure 2:**
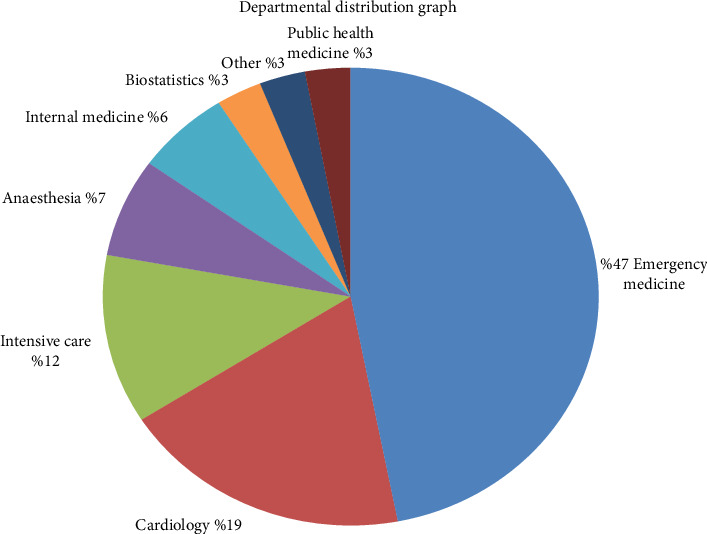
Distribution of the main authors by specialization.

The branch or specialization of the first author was recorded for each article. Emergency medicine was the leading specialization, contributing to nearly half of the articles, followed by cardiology (19%), intensive care (12%), and anesthesiology (7%). Specializations such as pediatrics, cardiovascular surgery, and general surgery were grouped under the category “other,” each comprising 1% of the total (Figure 2).

The 10 most-cited articles were ranked according to their Field-Weighted Citation Impact (FWCI), as recorded in the Scopus index. The FWCI indicates how frequently an article is cited compared with similar works. The article with the highest FWCI originated in Norway, whereas seven of the top 10 articles were authored in the United States. The article titled “Predicting Survival from Out-of-Hospital Cardiac Arrest: A Graphic Model” could not be ranked by the FWCI because the metric was introduced after 1996; therefore, it was placed at the bottom of the list (Table 1).

**Figure 3:**
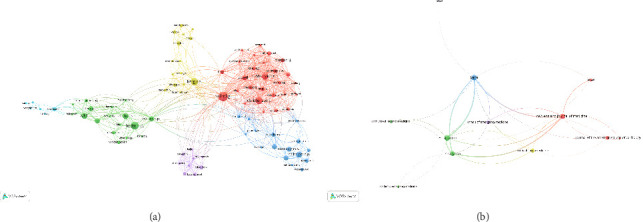
Relationship network map of authors and journals.

The interaction network among the 10 most active authors was analyzed. Notable first authors of highly cited articles include Lars Wik, Benjamin S. Abella, and Alfred Hallstrom. Although Graham Nichol was not the first author in the analyzed articles, he had the most extensive interaction network (Figure 3(a)).

When examining journals that published the top 100 articles, the New England Journal of Medicine (NEJM) published 25 articles, followed by the Journal of the American Medical Association (JAMA) [21], Resuscitation [15], and Circulation [15]. Circulation ranked first, with 509 citations, followed by resuscitation with 458 citations. The Annals of Emergency Medicine, JAMA, and NEJM received 257, 245, and 216 citations, respectively. The overlap between the journals that published the most articles and those that received the highest number of citations was notable (Figure 3(b)).

**Figure 4:**
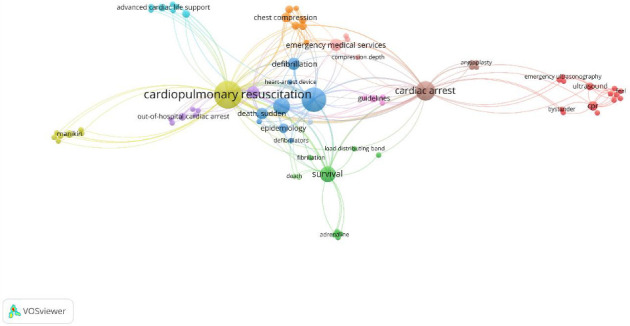
Most frequently used keywords' relationship.

The authors' keywords were analyzed, and the top 10 most frequently used terms were identified. “CPR” ranked first, accounting for over one-third of the top 10 keywords, followed by “heart arrest.” Other common terms included “cardiac arrest” and “resuscitation” (Figure 4).

**Figure 5:**
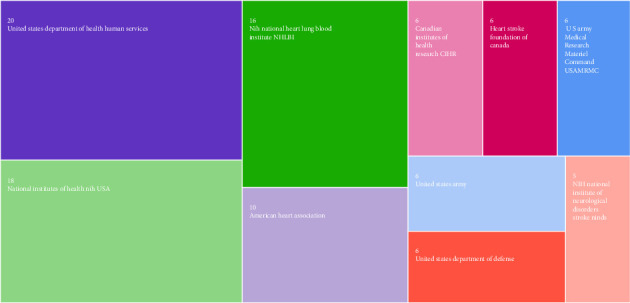
Top contributing organizations.

Among the top 10 organizations contributing to the articles, eight were based in the USA and two were from Canada. The top four organizations were all from the USA (Figure 5). Furthermore, 18 publications were funded by the USA military and 2 were funded by the Canadian military.

## 4. Discussion

Bibliometric analysis provides a snapshot of the current literature and serves as a roadmap for future research. In our study, we examined articles with high citation counts to evaluate their scientific contributions rather than to provide direct guidance for clinical practice. Specifically, we analyzed the most impactful and highly cited articles in the field of CPR. The most significant periods of research and citation activity coincided with the publication of guidelines by international organizations, including the International Liaison Committee on Resuscitation (ILCOR), American Heart Association (AHA), and European Resuscitation Council (ERC). These guidelines, published at regular intervals, aim to standardize CPR practices and provide updated knowledge to researchers and healthcare professionals [14].

The establishment of the ILCOR and the publication of the first systematic CPR recommendations marked the beginning of a structured database in this field. In this study, we exclusively focused on original articles to identify the foundational research underlying these guidelines. Our findings revealed that all the top 10 most-cited articles were referenced in either the AHA or ERC guidelines. This highlights the mutually reinforcing relationship between the guidelines and original research, creating a positive feedback loop that advances the literature. This result underscores the appropriateness of focusing solely on original articles in this analysis.

Key contributors to the CPR literature include organizations and journals predominantly based in the United States and Europe. Notable examples are the AHA, NEJM (Journal Impact Factor [JIF] 158.5), JAMA (JIF 120.7), and Circulation (JIF 37.8), as well as the ERC and Resuscitation (JIF 6.5). All first authors of the top 10 most-cited articles were affiliated with institutions in the United States, Canada, and Europe. This reflects the significant influence of geographically concentrated organizations and associations in shaping CPR research [15]. An analysis of citation networks within European countries also suggests that unifying these nations under the ERC and leveraging high-impact journals foster research development and collaboration.

Although NEJM and JAMA published the most articles, circulation and resuscitation received significantly more citations. These findings suggest that guidelines published every 5 years might amplify the citation impact of specific journals [16, 17].

Temporal analysis of the top 100 articles revealed an increase in publications after 2000, with a notable concentration of highly cited articles between 1997 and 2005. Milestones during this period included the ILCOR's establishment in 1992, the publication of the first detailed CPR recommendations in 1997, an AHA-led international CPR conference in 1992 with participation from 58 countries, and the release of ERC guidelines in 2001 and 2005. These developments fueled global interest in CPR and contributed to foundational research [18, 19].

When examining the journals included in our top 100 list, we identified three journals specific to the field of emergency medicine. Of the six articles published in these journals, five focused on out-of-hospital cardiac arrest (OHCA) and one addressed echocardiographic evaluation in patients with in-hospital cardiac arrest (IHCA). The remaining 94 articles were published in general medical journals and were not exclusive to emergency medicine. Notably, these articles were published in 14 journals. It is striking that the majority of articles were published in nonemergency medicine journals, despite the prominence of emergency physicians as first authors. We attribute this trend to two primary factors: the preference for publication in high-impact journals to enhance visibility and citation potential, and the broad relevance of the subject matter to multiple medical disciplines. In two separate bibliometric studies focusing on ultrasound use and trauma-oriented research in emergency medicine, resuscitation-related articles demonstrated significantly higher citation counts [20, 21]. This observation underscores the multidisciplinary nature of and widespread interest in CPR research.

A study by Dippenaar et al., which evaluated the top 100 most-cited articles in CPR research, noted a declining trend in annual citation rates over time. It also highlighted that articles published in high-impact journals were less affected by this decline [22]. However, in our analysis, we observed that citation counts decreased with the age of the article regardless of the journal's impact factor. In the top 100 most-cited articles, citation activity peaked in 2015, followed by a consistent decline. A similar trend was observed in the top 10 most-cited articles, further supporting this pattern. To contextualize this finding, note that Dippenaar et al.'s study included reviews, book chapters, and guidelines, which inherently have longer citation lifespans. Their analysis concluded in 2010, a period during which influential studies from the early 2000s continued to accumulate citations. By contrast, our study encompassed more recent articles, many of which have not yet reached the peak of their citation trajectory. Tahamtan, Safipour Afshar, and Ahamdzadeh also reported that citation rates generally decline with time, independent of other factors, which aligns with our findings [23]. This evidence underscores the importance of selecting contemporary topics to maximize an article's citation potential. Callaham, Wears, and Weber further emphasized that citations of prior research are a key determinant of an article's future citation impact [24].

Graham Nichol was the most prolific and cited author in our analysis. Similarly, in a bibliometric study by Jia et al., Graham Nichol ranked first among the most productive authors in CPR research [25]. Interestingly, Graham Nichol's extensive contributions, spanning 13 of the top 100 articles, solidified his influence despite not being the first author of any of the top articles. This impact is likely driven by his publications in high-impact journals, supported by an impressive h-index of 93 in the Scopus database [24].

First authors, such as Lars Wik, Benjamin S. Abella, and Alfred Hallstrom, who contributed to the most-cited articles, also demonstrated substantial academic influence. An analysis of the work of the four most influential authors revealed that most of their academic output focused on resuscitation, underscoring the value of specialization. This finding serves as a motivational example for early career researchers aiming to make significant contributions within a focused field.

Keyword analysis of the most-cited articles revealed a progressive increase in the use of terms such as “CPR” and “cardiac arrest,” particularly after the 2000s, coinciding with the publication of regular guidelines and the expansion of CPR literature. Conversely, terms like “defibrillation” and “sudden death” have gradually declined in prominence, reflecting changes in research priorities and guideline terminology over time [25, 26]. All these show the importance of using current keywords as well as the main keywords of the subject for authors who want to have a higher number of citations to be more effective authors.

An analysis of the funding sources for the most-cited articles revealed that over 80% of these publications were supported by organizations. Notably, the alignment between the country producing the highest number of cited articles and that providing the most funding underscores the strong relationship between scientific contributions and national support for research [15]. In this context, it is significant that more than half of these articles received funding from USA public health institutions. Compared with private organizations, state and university institutions provided the majority of funding. While the USA National Department of Health and Human Services accounted for the largest share of funding, military organizations supported an equal number of articles. Together, the USA and Canadian military institutions funded approximately 20% of the publications. This observation highlights the prominent role of military organizations in the field of resuscitation among primary funding entities [27]. Given the heightened risk of cardiac arrest among military personnel, these organizations demonstrate a strategic focus on reducing mortality through investments in academic research [28].

Unlike traditional analyses, our study employed bibliometric software to identify trends in CPR research and foundational work in this field despite some inherent limitations. A primary limitation of bibliometric studies is the exclusion of recently published high-quality articles that have not yet received sufficient citations.

## 5. Conclusion

Bibliometric analyses of CPR are relatively scarce. Our study represents one of the most comprehensive and frequently cited contributions in this domain. By evaluating prior research on CPR, we identified key milestones and emerging trends in this area. We believe that this analysis of the most impactful articles and their authors provides a valuable foundation for researchers, particularly early career academics who aspire to contribute to this field.

## Figures and Tables

**Table 1:** Top 10 authors and most-cited publications.

Country	Authors	Title	FWCI	Cited by	Source	Year
Norway	Wik, Lars	Quality of cardiopulmonary resuscitation during out-of-hospital cardiac arrest	68.34	1096	JAMA	2005
USA	Abella, Benjamin S.	Quality of cardiopulmonary resuscitation during in-hospital cardiac arrest	65.00	1058	JAMA	2005
USA	Hallstrom, Alfred	Public-access defibrillation and survival after out-of-hospital cardiac arrest	64.31	986	New England Journal of Medicine	2004
USA	Valenzuela, Terence D.	Outcomes of rapid defibrillation by security officers after cardiac arrest in casinos	53.34	1194	New England Journal of Medicine	2000
USA	Kudenchuk, Peter J.	Amiodarone for resuscitation after out-of-hospital cardiac arrest due to ventricular fibrillation	43.28	732	New England Journal of Medicine	1999
Denmark	Wissenberg, Mads	Association of national initiatives to improve cardiac arrest management with rates of bystander intervention and patient survival after out-of-hospital cardiac arrest	42.63	894	JAMA	2013
USA	Caffrey-Villari, Sherry L.	Public use of automated external defibrillators	41.14	706	New England Journal of Medicine	2002
Canada	Dorian, Paul	Amiodarone as compared with lidocaine for shock-resistant ventricular fibrillation	31.11	639	New England Journal of Medicine	2002
Netherlands	de Vreede-Swagemakers, Jacqueline J.M.	Out-of-hospital cardiac arrest in the 1990s: a population-based study in the Maastricht area on incidence, characteristics and survival	4.40	701	Journal of the American College of Cardiology	1997
USA	Larsen, Mary Pat	Predicting survival from out-of-hospital cardiac arrest: a graphic model		975	Annals of Emergency Medicine	1993

## Data Availability

All data generated or analyzed during this study are included in this published article. Data and materials are accessible and can be shared when necessary.
